# Gene expression of kynurenine pathway enzymes in depression and following electroconvulsive therapy

**DOI:** 10.1017/neu.2024.34

**Published:** 2024-10-17

**Authors:** Karen M. Ryan, Myles Corrigan, Therese M. Murphy, Declan M. McLoughlin, Andrew Harkin

**Affiliations:** 1 Trinity College Institute of Neuroscience, Trinity College Dublin, Dublin, Ireland; 2 Department of Psychiatry, St. Patrick’s University Hospital, Trinity College Dublin, Dublin, Ireland; 3 Neuropsychopharmacology Research Group, School of Pharmacy and Pharmaceutical Sciences & Trinity College Institute of Neuroscience, Trinity College, Dublin, Ireland; 4 School of Biological, Sports and Health Sciences, Technological University Dublin, Dublin, Ireland

**Keywords:** Depression, electroconvulsive therapy, glucocorticoid receptor, interleukin-6, kynurenine

## Abstract

**Objective:**

This study aimed to investigate changes in mRNA expression of the kynurenine pathway (KP) enzymes *tryptophan 2, 3-dioxygenase* (*TDO*), *indoleamine 2, 3-dioxygenase 1* and 2 (*IDO1*, *IDO2*), *kynurenine aminotransferase 1* and 2 (*KAT1, KAT2*), *kynurenine monooxygenase* (*KMO*) and *kynureninase* (*KYNU*) in medicated patients with depression (*n* = 74) compared to age- and sex-matched healthy controls (*n* = 55) and in patients with depression after electroconvulsive therapy (ECT). Associations with mood score (24-item Hamilton Depression Rating Scale, HAM-D24), plasma KP metabolites and selected glucocorticoid and inflammatory immune markers known to regulate KP enzyme expression were also explored.

**Methods:**

HAM-D24 was used to evaluate depression severity. Whole blood mRNA expression was assessed using quantitative real-time polymerase chain reaction.

**Results:**

*KAT1, KYNU* and *IDO2* were significantly reduced in patient samples compared to control samples, though results did not survive statistical adjustment for covariates or multiple comparisons. ECT did not alter KP enzyme mRNA expression. Changes in *IDO1* and *KMO* and change in HAM-D24 score post-ECT were negatively correlated in subgroups of patients with unipolar depression (*IDO1* only), psychotic depression and ECT responders and remitters. Further exploratory correlative analyses revealed altered association patterns between KP enzyme expression, KP metabolites, *NR3C1* and *IL-6* in depressed patients pre- and post-ECT.

**Conclusion:**

Further studies are warranted to determine if KP measures have sufficient sensitivity, specificity and predictive value to be integrated into stress and immune associated biomarker panels to aid patient stratification at diagnosis and in predicting treatment response to antidepressant therapy.

## Graphical abstract

**Figure:**
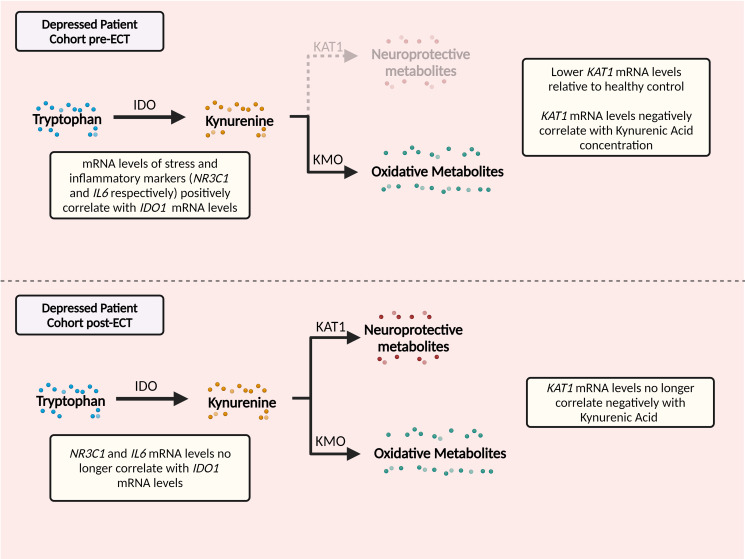

## Introduction

The kynurenine pathway (KP) is a major route through which the essential amino acid tryptophan is metabolised and activated in times of stress and immune activation.

Initially the conversion of tryptophan to kynurenine requires induction of either of three rate limiting enzymes, tryptophan 2, 3-dioxygenase (TDO) or indoleamine 2, 3-dioxygenase 1 and 2 (IDO1 and IDO2). TDO/IDO 1 and 2 metabolise tryptophan to kynurenine, which is subsequently converted to either kynurenic acid (KYNA) by kynurenine aminotransferase (KAT of which there are four subtypes) or 3-hydroxykynurenine by kynurenine monooxygenase (KMO). 3-Hydroxykynurenine (3-HK) is further metabolised to either anthranillic acid (AA) or 3-hydroxyanthranilic acid (3-HAA) by kynureninase (KYNU), giving rise to acetyl-CoA, or to the unstable intermediate, 2-amino-3-carboxymuconate by 3-hydroxyanthranilic acid 3, 4-dioxygenase (3-HAO). This metabolite is further enzymatically converted to picolinic acid, or non-enzymatically transformed to quinolinic acid (QUIN), the precursor for nicotinamide adenine dinucleotide (NAD+) (Lugo-Huitrón *et al*., 2013; Vécsei *et al*., 2013). 3-HAA can also be metabolised to picolinic acid by aminocarboxymuconate-semialdehyde decarboxylase (ACMSD). KAT can also convert 3-HK to xanthurenic acid [for a schematic of the pathway see O’Farrell and Harkin (2017)].

KP activation has been proposed to play a role in depression (Réus *et al*., 2015; Myint and Halaris, 2022), with reports of reduced circulating concentrations of tryptophan, an increased tryptophan breakdown index (kynurenine/tryptophan) and decreased KYNA concentrations in depressed patients in comparison to healthy controls (Schwieler *et al*., 2016; Allen *et al*., 2018; Arnone *et al*., 2018; Doolin *et al*., 2018; Correia and Vale, 2022). Activation of the pathway, coupled with reduced tryptophan, has been observed in depression occurring secondary to exogenous administration of cytokines such as IFN-α and IL-2 (Raison *et al*., 2010). The KP is generally divided into neurotoxic and neuroprotective branches, with greater activation of the neurotoxic branch typically reported in depression (Savitz, 2020). The neuroprotective arm of the KP is driven by the KAT enzymes, which catalyse the conversion of kynurenine to KYNA. The neurotoxic arm is driven by KMO and KYNU, which catalyse the conversion of kynurenine to the downstream metabolites 3-HK, 3-HAA and QUIN (Stone *et al*., 2013). Studies have reported that both IDO and TDO are overactive in depression, suggesting that inhibiting these enzymes might be useful for treating depression (Qin *et al*., 2018). However, increased IDO is also suggested to have a protective effect through a compensatory anti-inflammatory reflex system (CIRS), which acts to attenuate immunoinflammatory responses (Maes *et al*., 2012).

We have previously reported that tryptophan and kynurenine metabolite concentrations (AA, 3-HAA, picolinic acid, KYNA and xanthurenic acid)] and KYNA/kynurenine and KYNA/QUIN ratios are reduced in medicated depressed patients in comparison to healthy controls (Ryan *et al*., 2020). Improvements in mood score following a course of electroconvulsive therapy (ECT) and treatment response were correlated with increased kynurenine, 3-HK, 3-HAA, QUIN and kynurenine/tryptophan in unipolar depressed patients. These results suggest that ECT may mobilise the KP since a moderate association between selected metabolites and treatment response was evident in unipolar depressed patients.

Assessments of KP activity to date have largely relied on measuring tryptophan and kynurenine metabolite concentrations. Few have examined and reported changes in the mRNA expression of the KP enzymes (Hughes *et al*., 2012; Doolin *et al*., 2018; Brown *et al*., 2022). Assessment of KP enzyme expression is important considering induction of TDO or IDO enzymatic activity by glucocorticoids/cytokines respectively are reliant on de novo synthesis of the enzymes. In individuals with depression, increased *KAT1* and *KAT2* mRNA expression was reported in the anterior cingulate cortex (ACC), including in those patients with psychotic and non-psychotic depression compared to matched controls (Brown *et al*., 2022). The results from the Sequenced Treatment Alternatives to Relieve Depression (STAR*D) study (*n* = 1953) suggested that a common single nucleotide polymorphism in *IDO2* is linked to the response to treatment with the antidepressant citalopram (Cutler *et al*., 2012).

Glucocorticoid release from the adrenal glands, associated with stress-induced activation of the hypothalamic pituitary adrenal (HPA) axis, can induce TDO because the promoter region of TDO harbours glucocorticoid response elements. Moreover, IDO may be induced in response to stress through sympathoadrenal medullary (SAM) system-associated β-adrenergic receptor activation of immune cells, resulting in the release of pro-inflammatory cytokines, including interleukin-1 beta (IL-1β), IL-6 and interferon (IFN)-γ (Elenkov *et al*., 2000). Moreover, Doolin and co-workers reported a positive correlation between plasma QUIN concentrations, with concentrations of the inflammatory marker C-reactive protein and whole blood *IDO1* mRNA expression in patients with major depressive disorder (MDD) (Doolin *et al*., 2018). Thus, measurement of KP enzyme expression is important considering that induction of enzymatic activity by glucocorticoids/cytokines are reliant on de novo synthesis of the enzymes.

In this study, we build on previous reported findings (Ryan *et al*., 2020) to investigate mRNA expression of KP enzyme targets in samples from medicated patients with depression versus age- and sex-matched healthy controls and in patients with depression after ECT, taking account of co-variables including heterogenous psychopathology. We hypothesised that there would be a reduction in mRNA expression of KP enzymes in patient blood compared to that from controls, in line with our previous study report. In addition, we also examined associations between KP enzyme mRNA expression and mood score. We also performed exploratory analyses to assess associations between KP enzyme mRNA expression with stress and immunological markers. We hypothesised that stress and immunological markers commonly linked to the pathophysiology of depression would correlate with KP enzyme mRNA levels and be subject to change in patients with depression after ECT. Further exploratory correlation analyses were undertaken with plasma KP metabolites and the mRNA expression of selected glucocorticoid markers [glucocorticoid receptor (*NR3C1*), the GR co-chaperone protein *FK506 binding protein* (*FKBP5*) and *glucocorticoid-induced leucine zipper* (*GILZ*), a key molecule in glucocorticoid biology that mediates the downstream anti-inflammatory effects of the glucocorticoid receptor] and inflammatory immune markers [*tumour necrosis factor alpha* (*TNF-α*) and *IL-6*)].

## Material and methods

### Participants

This study was approved by St Patrick’s University Hospital Research Ethics Committee and carried out in line with the Declaration of Helsinki (World Medical Association, 2013). Written informed consent was provided by all participants. Medicated depressed patients were recruited in St Patrick’s Mental Health Services (http://www.stpatricks.ie/) as part of the EFFECT-Dep (Enhancing the Effectiveness of ECT in Severe Depression; ISRCTN23577151) Trial, a real-world, pragmatic, patient- and rater-blinded, non-inferiority trial of patients with major depression carried out to assess the effects of twice-weekly moderate-dose bitemporal (1.5× seizure threshold) and high-dose unilateral (6× seizure threshold) ECT (Semkovska *et al*., 2016). ECT was administered twice weekly with patients maintained on their usual pharmacotherapy during the course of treatment. Methohexital (0.75 mg/kg–1.0 mg/kg) was used for anesthesia and succinylcholine (0.5 mg/kg–1.0 mg/kg) was used as muscle relaxant. Inclusion criteria for this study were as follows: >18 years old; referred for ECT for treatment of a major depressive episode (unipolar and bipolar), as diagnosed by the Structured Clinical Interview for DSM-IV Axis I Disorders (First *et al*., 1996); pre-treatment Hamilton Depression Rating Scale 24-item version (HAM-D24) (Beckham and Leber, 1985) score≥21. Exclusion criteria were as follows: ECT or substance misuse in the previous six months; medically unfit for general anaesthesia; dementia or other axis I diagnosis. Healthy controls were recruited through local newspaper and social media advertisements.

We recorded demographic data and medical/treatment history for all participants. Depression severity and ECT response were assessed using the HAM-D24. Response was defined as a ≥60% reduction in HAM-D24 and a score ≤ 16 at end-of-treatment, while remission was defined as a ≥60% reduction in HAM-D24 and a score ≤ 10 maintained for two weeks.

Fasting peripheral blood samples (2.5 mL) were collected into PAXgene© Blood RNA tubes (PreAnalytix, Qiagen Ltd., Ireland) at 07:30–09:30 on the baseline (i.e., before the first ECT treatment) day and 1–3 days after the final ECT treatment and from controls on assessment days between 07:30 and 09:30, per manufacturer’s guidelines. Tubes were stored at room temperature for 24 h, -20°C for 24 h, followed by long-term storage at –80 °C. We excluded any participants with a chronic immune disorder or neurological disorder from molecular analyses. In total, 74 patients with depression and 55 controls were included in these analyses.

### Quantitative real-time polymerase chain reaction (qPCR)

qPCR was carried out as previously described (Ryan *et al*., 2019), with slight modifications. Briefly, a PAXgene Blood RNA kit (PreAnalytix) was used to extract whole blood mRNA and a high capacity cDNA archive kit (Applied Biosystems, UK) was used to carry out reverse transcription of samples. qPCR was performed using a StepOnePlus^TM^ instrument (Applied Biosystems) with TaqMan® Gene Expression Assays (*IDO1*, Hs00984148_m1; *IDO2*, Hs01589373_m1; *KMO*, Hs00175738_m1; *KYNU*, Hs011114099_m1; *KATI*, Hs00187858_m1; *KATII*, Hs00212039_m1; *TDO*, Hs00194611_gH; *GAPDH*, Hs02758991_g1) and TaqMan® Fast Advanced Master Mix (Applied Biosystems). The cycling conditions consisted of an initial polymerase activation step of 95 °C for 20 s followed by 50 cycles of 95 °C for 1 s and 60 °C for 20 s. We calculated relative quantification (RQ) levels using the comparative CT method (Schmittgen and Livak, 2008) against a calibrator sample from a healthy volunteer, not included in the study, after normalisation to *GAPDH*.

The method and assay IDs for *NR3C1*, *FKBP5*, *GILZ, IL-6* and *TNF-α* were reported previously (Ryan and McLoughlin, 2019; Ryan *et al*., 2020).

### Tryptophan and KP metabolite concentrations

Tryptophan and KP metabolite concentration measurements were reported previously (Ryan *et al*., 2020).

### DNA methylation analysis

DNA methylation levels, at CpG sites annotated (based on closest transcriptional start site) to *KAT1*, *KMO*, *KYNU*, *IDO1* and *IDO2,* were obtained from a previously published methylome-wide association study of a self-reported history of depression (Crawford *et al*., 2018). Briefly, DNA methylation levels were quantified using the Illumina Infinium HumanMethylation450 BeadChip (“Illumina 450K array”) array. A full description of data quality control and normalisation methods were reported previously (Crawford *et al*., 2018). DNA methylation scores for the following CpG sites (cg13263723 (*KYNU*); cg21542308 (*IDO2*); cg08465774 (*IDO1)*; cg00606312 (*KMO*) were available in the dataset.

### Statistical analysis

All statistical analyses (except for the DNA methylation analysis) were performed using SPSS, version 26 (IBM Corporation, NY, USA). Data were tested for normality using Q-Q plots and a Shapiro-Wilk test and subsequently log-transformed where indicated. Baseline clinical and demographic characteristics are shown as means with SD or number (%) per group where appropriate and categorical data were analysed using Chi-square (*χ* ^2^) tests.

**Figure 1.**
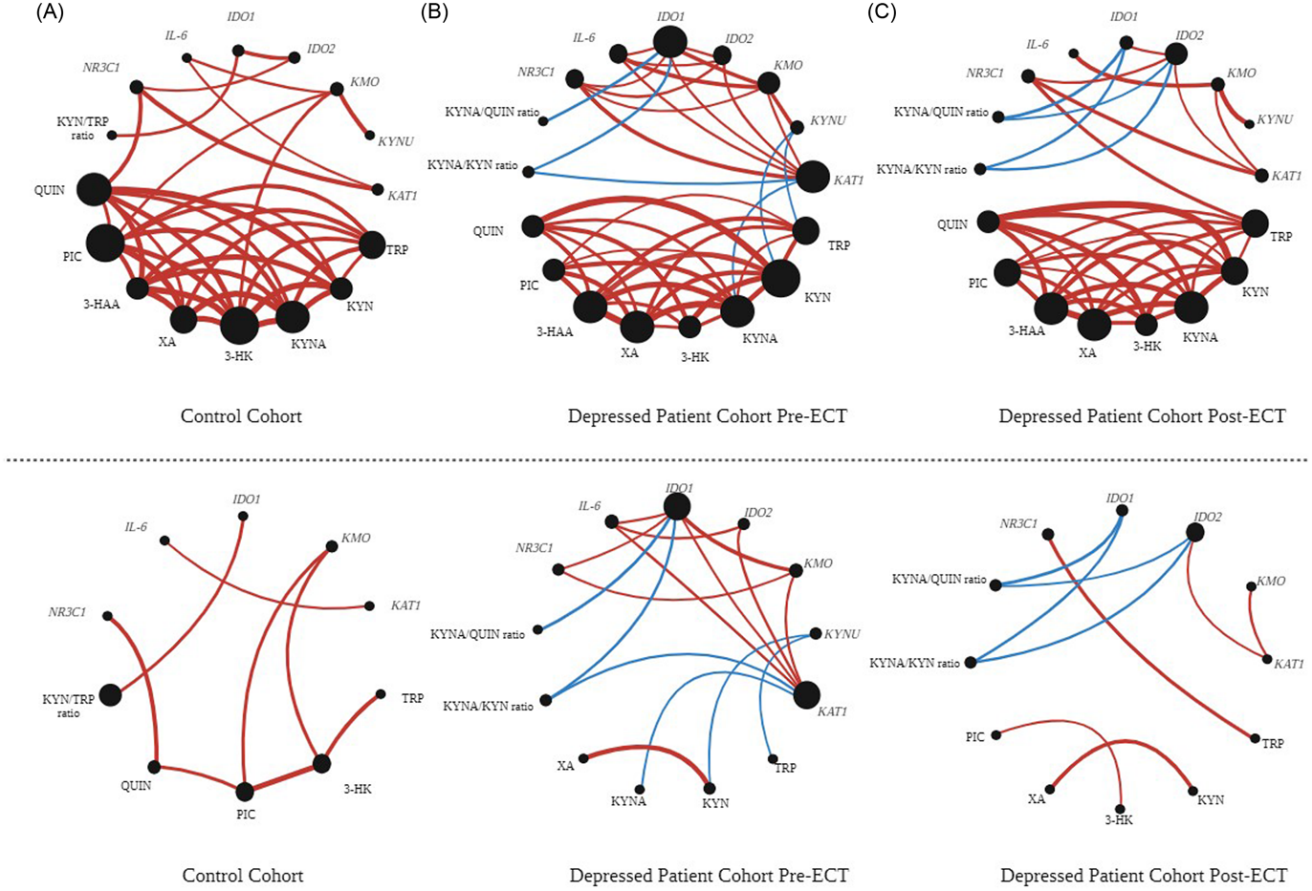
**Correlation-based representations of associations between tryptophan metabolite concentrations and mRNA expression of kynurenine pathway enzymes, *IL-6* and the glucocorticoid receptor in whole blood** in (A) a healthy control cohort, (B) a depressed patient cohort pre-ECT and (C) the same depressed patient cohort post-ECT. While a wider panel of inflammatory and stress markers were investigated, *IL-6* and *NR3C1* were selected as representative markers for clarity of the schematic. These additional correlations are available in Supplementary Table 8. *Top panel*: all significant correlations are included. The size of each node is proportional to the number of correlations at that node. The width of each line is proportional to the strength of the correlation (quantified by the statistical rho value). Red lines correspond to positive correlations and blue lines correspond to negative correlations. *Bottom panel*: Significant correlations common between all groups are removed in order to highlight differences between the groups. Node sizes are adjusted accordingly. Abbreviations: IDO, indolamine 2, 3-dioxygenase; KMO, kynurenine 3-monooxygenase; KYNU, kynureninase; KAT, kynurenine aminotransferase; TRP, tryptophan; KYN, kynurenine; 3-HK, 3-hydroxykynurenine; XA, xanthurenic acid; 3-HAA, 3-hydroxyanthranillic acid; KYNA, kynurenic acid; PIC, picolinic acid; QUIN, quinolinic acid; GR, glucocorticoid receptor.

RQ data were analysed using general linear models. We adjusted for potential variance owing to age, sex, body mass index (BMI; kg/m^2^) and smoking status as these have previously been associated with changes in the KP (Theofylaktopoulou *et al*., 2013; Mangge *et al*., 2014; Favennec *et al*., 2015; Raheja *et al*., 2015; de Bie *et al*., 2016). Smoking status was dichotomised into current versus non-smoker. Adjustment was also made for educational attainment as this was significantly different between groups. For pre-/post-ECT analyses, depression polarity, depression severity at baseline and presence of psychosis were included as covariates where appropriate. Data not normally distributed after log10 transformation were analysed using non-parametric methods (Mann-Whitney U or Wilcoxon Signed Rank test). Correlation analyses were carried out using either Pearson’s product-moment correlation coefficient (Pearson’s *r*) or Spearman’s rank correlation coefficient rho (Spearman’s *ρ*). Representations of associations between mRNA expression of KP enzymes and tryptophan metabolite concentrations, *IL-6* and *NR3C1* in whole blood in healthy controls, depressed patients pre-ECT and depressed patients post-ECT are shown in Figure 1. All significant correlations are included. The size of each node is proportional to the number of correlations at that node, while the width of each line is proportional to the strength of the correlation (quantified by the statistical rho value).

Statistical analyses for the DNA methylation analysis were performed using R (version 3.2.1). Linear regression was used to examine differences in DNA methylation scores [reported as change in beta value] between individuals with a self-reported history of depression and individuals without a history of depression at CpG sites (cg13263723 (*KYNU*); cg21542308 (*IDO2*); cg08465774 (*IDO1)*; cg00606312 (*KMO*)), controlling for potential confounders (history of an inflammatory disorder, age, gender, anti-depressant use, chip and estimated blood cell composition).

Data are expressed as means with standard deviation (SD). Differences with a *p*-value<0.01, consistent with analysis of five targets, namely *KAT1*, *KMO*, *KYNU*, *IDO1* and *IDO2*, were deemed statistically significant to account for multiple comparisons (Bonferroni correction).

## Results

### Demographics and clinical characteristics

Demographic and clinical characteristics of patients with depression and healthy controls are shown in Table 1. Groups differed with respect to BMI, smoking and education, with the patient group having a higher BMI, a higher percentage of smokers and a lower level of educational attainment compared to controls.

**Table 1.** Demographic and clinical characteristics of participants

	Depressed Baseline/Pre-ECT (*n* = 74)	Controls (*n* = 55)	Statistical test
Age, years	56.43 (14.34)	53.94 (12.50)	*t* = 1.08, *p* = 0.28
Sex, No. (%)			
Male	27 (36.49)	20 (36.36)	*χ* ^*2*^ = 0.0002, *p* = 0.99
Female	47 (63.51)	35 (63.64)	
BMI	27.09 (5.22)	24.64 (3.18)	*t* = 3.06, *p* = 0.001
Smokers, No. (%)	31 (41.89)	4 (7.27)	*χ* ^*2*^ = 9.19, *p* = 0.002
Education, No. (%)			
Primary	14 (18.92)	3 (5.45)	*χ* ^*2*^ = 30.35, *p*<0.001
Secondary	41 (55.41)	11 (20)	
Tertiary & Quaternary	19 (25.68)	41 (74.55)	
Bipolar depression, No. (%)	18 (24.32)		
Psychotic depression, No. (%)	19 (25.68)		
Baseline HAM-D24	30.05 (5.92)	3.11 (2.56)	*U* = 0.000, *p*<0.001
Post-ECT HAM-D24	11.05 (8.34)		
Electrode placement, No. (%)			
Unilateral	31 (41.89)		
Bitemporal	43 (58.11)		
Number of ECT sessions	8.26 (2.41)		
Responders, No. (%)	45 (60.81)		
Remitters, No. (%)	39 (52.70)		
Medications, No. (%) taking			
SSRI	22 (29.73)		
SNRI	35 (47.30)		
TCA	15 (20.27)		
MAOI	1 (1.35)		
Mirtazapine	29 (39.19)		
Bupropion	2 (2.70)		
Lithium	30 (40.54)		
Anticonvulsant	26 (35.14)		
Antipsychotics	58 (78.38)		
Benzodiazepines	45 (60.81)		
Non-benzodiazepine hypnotics	36 (48.65)		
Buspirone	1 (1.35)		
Agomelatine	5 (6.76)		
Trazadone	3 (4.05)		

Data are presented as means with standard deviations (SD) or number (%) per group where appropriate, unless otherwise stated.

BMI, body mass index; ECT, electroconvulsive therapy; HAM-D24, Hamilton depression rating scale, 24-item version; MAOI, monoamine oxidase inhibitor; SNRI, serotonin-norepinephrine reuptake inhibitor; SSRI, selective serotonin reuptake inhibitor; TCA, tricyclic antidepressant.

### KP enzyme mRNA levels in patients with depression and healthy controls

The mRNA expression of KP enzymes *KAT1, KMO, KYNU, IDO1* and *IDO2* in whole blood samples from medicated patients with depression compared to controls are shown in Table 2. For this study, *KAT1* was chosen as representative of the KAT subtypes. The mRNA expression of *KAT2* and *TDO* was also measured but was below the limits of detection. The unadjusted statistical analysis shows that *KAT1, KYNU* and *IDO2* levels were significantly lower in the patient group compared to controls (*p* < 0.01). There was no statistically significant difference between groups following adjustment for covariates.

**Table 2.** KP enzyme mRNA levels in healthy controls compared to patients with depression

	Healthy controls	Depressed Patients	Unadjusted Statistics	Adjusted Statistics †
*KAT1*	2.14 ± 0.74	1.83 ± 0.59	F_1,127_ = 7.64, ***p* = 0.007**	F_1,98_ = 4.73, *p* = 0.03
*KMO*	2.19 ± 0.80	1.87 ± 0.79	F_1,125_ = 5.71, *p* = 0.018	F_1,97_ = 0.25, *p* = 0.62
*KYNU*	0.78 ± 0.29	0.60 ± 0.30	*U* = 2621.50, ***p* = 0.00007**	
*IDO1*	4.69 ± 4.22	4.04 ± 3.63	F_1,122_ = 1.23, *p* = 0.27	F_1,94_ = 1.49, *p* = 0.23
*IDO2*	1.74 ± 1.15	1.08 ± 0.70	*U* = 2636, ***p* = 0.00007**	

Data are expressed as means ± SD.

*KAT1*: depressed *n* = 74, controls *n* = 55; *KMO*: depressed *n* = 73, controls *n* = 54; *KYNU*: depressed *n* = 71, controls *n* = 52; *IDO1:* depressed *n* = 70, controls *n* = 54; *IDO2*: depressed *n* = 70, controls *n* = 53.

*KAT1*, *KMO* and *IDO1* data were log_10_ transformed prior to statistical analysis. †Adjusted for age, sex, BMI, smoking, educational attainment.

We also compared KP enzyme expression in males and females (Supplemental Table 1). *KAT1* significantly differed between female controls and female patients with depression, *KYNU* significantly differed between male controls and male patients with depression and *IDO2* significantly differed between male controls and male patients with depression and female controls and female patients with depression. No significant difference was noted between males and females within the control or depressed groups.

### KP enzyme mRNA levels in patients with depression pre- and post-ECT

Table 3 shows the mRNA levels of KP enzymes in blood samples from medicated patients with depression before (pre-) and after (post-) ECT. No significant changes were noted at *p* < 0.01 in either the unadjusted or adjusted statistical analyses.

**Table 3.** Depressed pre- and post-ECT

	Pre-ECT	Post-ECT	Unadjusted Statistics	Adjusted Statistics
*KAT1*	1.83 ± 0.59	1.86 ± 0.57	F_1,73_ = 0.27, *p* = 0.61	F_1,62_ = 0.006, *p* = 0.94
*KMO*	1.87 ± 0.79	2.00 ± 0.90	F_1,72_ = 1.44, *p* = 0.23	F_1,62_ = 0.85, *p* = 0.36
*KYNU*	0.60 ± 0.30	0.67 ± 0.30	*U* = 1539.50, *p* = 0.13	
*IDO1*	4.04 ± 3.63	4.19 ± 5.15	F_1,69_ = 0.53, *p* = 0.47	F_1,59_ = 2.73, *p* = 0.10
*IDO2*	1.08 ± 0.70	1.46 ± 1.27	*U* = 1522.50, *p* = 0.10	

Data are expressed as means ± SD.

*KAT1*: *n* = 74; *KMO*: *n* = 73; *KYNU*: *n* = 71; *IDO1*: *n* = 70; *IDO2*: *n* = 70.

*KAT1*, *KMO*, *IDO1* were log_10_ transformed prior to statistical analysis.

†Adjusted for age, sex, BMI, smoking, education, baseline HAM-D24, electrode placement, polarity, psychosis.

Subgroup analyses showed no difference between patients with unipolar vs. bipolar depression, psychotic vs. non-psychotic depression, ECT-responders vs. non-responders, ECT remitters versus non-remitters, or male versus female patients (Supplemental Tables 2–6, respectively).

### Correlations between KP enzymes and mood score

Moderate negative correlations were noted between the change in *KMO* and change in HAM-D24 score following ECT in patients with psychotic depression and ECT remitters and responders (Table 4), indicating that an increase in *KMO* was associated with improvement in mood in these patient subgroups. Moderate negative correlations were also noted between the change in *IDO1* and change in HAM-D24 score following ECT in patients with unipolar depression, psychotic depression and ECT remitters and responders (Table 5), indicating that an increase in *IDO1* was associated with improved mood in these patient subgroups. Baseline *IDO1* levels and baseline HAM-D24 score were negatively correlated in non-responders and non-remitters following ECT, indicating that lower *IDO1* at baseline was associated with a lack of therapeutic response to ECT. There was a moderate negative correlation noted between baseline *IDO2* levels and baseline HAM-D24 score in patients with psychotic depression (Supplemental Table 7), indicating that lower *IDO2* at baseline was associated with more severe depressive symptoms in this patient subgroup, though caution is warranted owing to the small sample size included in this analysis (*n* = 18). There were no other significant correlations noted in patient subgroups with respect to *KMO, IDO1,* or *IDO2* and mood score at baseline or following ECT, or between *KAT1* or *KYNU* and baseline or post-ECT mood score (Supplemental Table 8 & 9, respectively).

**Table 4.** Correlations between *KMO* and HAM-D24 scores

	Baseline *KMO* & Baseline HAM-D24	Baseline *KMO* & ΔHAM-D24	Δ*KMO* & ΔHAM-D24
*Polarity*			
Unipolar (*n* = 55)	*ρ* = −0.077, *p* = 0.573	*ρ* = 0.151, *p* = 0.271	*ρ* = −0.195, *p* = 0.155
Bipolar (*n* = 17)	*r* = 0.038, *p* = 0.886	*r* = −0.378, *p* = 0.135	*r* = −0.581, *p* = 0.015
*Psychosis*			
Yes (*n* = 19)	*ρ* = −0.115, *p* = 0.638	*ρ* = −0.127, *p* = 0.605	*r* = −0.662, ***p* = 0.004**
No (*n* = 53)	*ρ* = −0.047, *p* = 0.734	*ρ* = 0.132, *p* = 0.347	*r* = −0.141, *p* = 0.314
*Response*			
Yes (*n* = 45)	*ρ* = 0.074, *p* = 0.630	*ρ* = −0.087, *p* = 0.570	*ρ* = −0.432, ***p* = 0.003**
No (*n* = 27)	*ρ* = −0.247, *p* = 0.206	*ρ* = 0.217, *p* = 0.278	*r* = 0.064, *p* = 0.749
*Remission*			
Yes (*n* = 39)	*ρ* = 0.120, *p* = 0.467	*ρ* = −0.150, *p* = 0.362	*ρ* = −0.405, ***p* = 0.011**
No (*n* = 33)	*ρ* = −0.289, *p* = 0.098	*ρ* = 0.160, *p* = 0.375	*ρ* = −0.069, *p* = 0.703

HAM-D24, Hamilton depression rating scale, 24-item version; ΔHAM-D24, change in Hamilton depression rating scale, 24-item version score.

**Table 5.** Correlations between *IDO1* and HAM-D24 scores

	Baseline *IDO1* & Baseline HAM-D24	Baseline *IDO1* & ΔHAM-D24	Δ*IDO1* & ΔHAM-D24
*Polarity*			
Unipolar (*n* = 52)	*ρ* = −0.106, *p* = 0.449	*ρ* = 0.126, *p* = 0.375	*ρ* = −0.293, ***p* = 0.035**
Bipolar (*n* = 17)	*ρ* = −0.193, *p* = 0.458	*ρ* = −0.106, *p* = 0.685	*ρ* = −0.015, *p* = 0.955
*Psychosis*			
Yes (*n* = 18)	*ρ* = −0.400, *p* = 0.100	*ρ* = 0.336, *p* = 0.172	*ρ* = −0.531, ***p* = 0.023**
No (*n* = 51)	*ρ* = −0.060, *p* = 0.674	*ρ* = 0.048, *p* = 0.740	*ρ* = −0.172, *p* = 0.229
*Response*			
Yes (*n* = 44)	*ρ* = 0.077, *p* = 0.618	*ρ* = 0.039, *p* = 0.800	*ρ* = −0.446, ***p* = 0.002**
No (*n* = 25)	*ρ* = −0.597, ***p* = 0.008**	*ρ* = 0.114, *p* = 0.588	*ρ* = −0.232, *p* = 0.265
*Remission*			
Yes (*n* = 38)	*ρ* = 0.111, *p* = 0.505	*ρ* = −0.010, *p* = 0.953	*ρ* = −0.422, ***p* = 0.008**
No (*n* = 31)	*ρ* = −0.442, ***p* = 0.011**	*ρ* = 0.178, *p* = 0.337	*ρ* = −0.124, *p* = 0.508

HAM-D24, Hamilton depression rating scale, 24-item version; ΔHAM-D24, change in Hamilton depression rating scale, 24-item version score.

### Correlations between KP enzyme mRNA and KP metabolites

Correlation analyses of mRNA levels of KP enzyme targets with KP metabolite concentrations and with mRNA expression of *NR3C1*, *FKBP5*, *GILZ*, *TNF-alpha* and *IL-6* revealed altered association patterns between the expression of KP enzymes, *NR3C1*, *IL-6* and KP metabolites in depressed patients pre- and post-ECT (Supplemental Table 10). Correlations between KP metabolite concentrations themselves were also assessed. These patterns are summarised in Figure 1.

Associations between the expression of KP enzymes in depressed patients are represented by six significant correlations between mRNA expression of KP enzymes – ranging from a weak positive correlation between *IDO1* and *KAT1* levels to a moderate-strong positive correlation between *IDO1* and *KMO* levels. This is compared with two such correlations in healthy controls and four in the post-ECT depressed cohort. In healthy controls, *IDO1* level positively correlated with the KYN/TRP ratio, which is expected as IDO1 catalyses the conversion of TRP to KYN. However, this was absent in patients with depression. In addition, *IDO1* levels and the KYNA/QUIN ratio were found to be negatively correlated in depressed patients both pre- and post-ECT. This correlation is not evident in healthy controls. *IDO1* levels also negatively correlated with the KYNA/KYN ratio in the depressed patient cohort; this correlation was absent in the healthy control group and persisted post-ECT in the depressed group. Further to this, the negative correlations between (1) *KAT1* mRNA and KYNA metabolite levels and (2) *KAT1* mRNA and the KYNA/KYN ratio were unique to the depressed patient group indicating an interesting disparity between whole blood *KAT1* expression and plasma KYNA concentrations. Correlations of *NR3C1* with *IDO2* and *KAT1* were common to healthy controls and depressed patients pre- and post-ECT; however, correlations between *NR3C1* with *IDO1* and *KMO* were unique to depressed patients pre-ECT. In the depressed patient group, *IL-6* mRNA levels correlated with *IDO1, IDO2, KMO* and *KAT1* mRNA levels, while in healthy controls correlations between *IL-6, KMO* and *KAT1* mRNA levels were identified. The correlation between *IL-6* and *KMO* persisted in the depressed patient cohort post-ECT. Differential association patterns between healthy controls compared to depressed patients both pre- and post-ECT are not apparent with KP metabolites when correlated to *NR3C1 or IL-6* mRNA levels (Figure 1).

### KP enzyme DNA methylation levels in individuals with a self-reported history of depression compared to controls

Considering the significantly reduced mRNA levels of *KAT1, KYNU* and *IDO2* observed in our clinically depressed cohort, we also assessed DNA methylation levels of four out of the five the KP enzyme genetic loci (*KMO*, *KYNU*, *IDO1* and *IDO2*) to examine their association with depression. Analysis of DNA methylation levels in whole blood samples from individuals with self-reported depression history compared to controls revealed significant hypermethylation at cg21542308 (*IDO2*) in the depression group (*p* < 0.01, Supplemental Table 11).

## Discussion

The results indicate significantly reduced *KAT1, KYNU* and *IDO2* mRNA levels in whole blood from patients with depression in comparison to healthy controls, though none of these results survived statistical adjustment for potential covariates and multiple comparisons. Results also show that ECT had no effect on KP enzyme mRNA expression, either in the depressed group as a whole or in subgroup analyses. There were, however, moderate negative correlations observed between the change in *KMO* and change in HAM-D24 score in subgroups of patients with psychotic depression and ECT remitters and responders, indicating that, at an individual level, as KMO expression increased mood score improved. Moreover, there were weak to moderate negative correlations between the change in *IDO1* and change in HAM-D24 score post-ECT in subgroups of patients with unipolar depression, psychotic depression and ECT responders and remitters, indicating that, at an individual level, as *IDO1* increased following ECT mood score improved in these patient subgroups. Baseline *IDO1* was negatively correlated with baseline HAM-D24 score in non-responders and non-remitters, indicating that lower *IDO1* levels were related to non-response and non-remission following ECT. Baseline *IDO2* also moderately negatively correlated with baseline HAM-D24 score in patients with psychotic depression and in non-remitters, indicating that lower *IDO2* levels were related to more severe depressive symptoms in this patient subgroup and non-remission following ECT. Overall, these results are in line with those previously reported for this cohort with regard to the KP (Ryan *et al*., 2020), where reduced activation is evident in depressed patients and pathway mobilisation is associated with improvement in mood.

Previous research has reported no change in whole blood *IDO1/2*, *KAT1*, *KMO* or *KYNU* mRNA levels in patients with MDD compared to controls (Hughes *et al*., 2012). However, in comparison to the cohort included in our study, the sample comprised a younger cohort of patients with MDD (average age = 42 years) and patients were receiving either monotherapy with selective serotonin reuptake inhibitors (SSRIs) or being treated with a dual acting antidepressant, while ∼ 30% were medication-free, with those receiving antipsychotics or mood stabilisers excluded. Further analysis of a subsequent cohort of depressed patients by Doolin *et al*. (2018) also reported no difference in *KAT1* or *IDO1* expression between controls and individuals with MDD, but as before this depressed cohort was young (average 33 years), with approximately one third of study participants medication free. Positive correlations between *IDO1* mRNA expression, QUIN and the kynurenine/tryptophan ratio were observed, while *KAT1* mRNA expression and depression scores were reported to be negatively correlated (Doolin *et al*., 2018). In contrast, increased *KAT1* and *KAT2* mRNA expression has been reported in the ACC of individuals with depression, including in those patients with psychotic and non-psychotic depression compared to matched controls (Brown *et al*., 2022). However, in comparison to the cohort included in the present study, the sample size was small and subjects were young (average age ∼ 42). Moreover, a subsequent study from the same group showed a significant difference in *KAT2* in the ACC between controls and patients with depression and in *KMO* between male and female controls, though no difference in *KMO* was noted between males and females with depression or between the control and depressed groups as a whole (Brown *et al*., 2024). In this study, we found no difference in any of the KP target enzyme mRNA levels between males and females in either depressed or healthy control groups.

As IDO is a rate-limiting enzyme of the KP, pathway activation is gauged by IDO activity. The fact that our correlation analyses showed a unique association between *IDO1* and *KMO* levels in the depressed patient cohort suggests that in depression induction of the KP is correlated to *KMO* level, and a branch of the KP associated with oxidative stress – an association which appears to be negated by ECT. To our knowledge, no study has compared peripheral and central levels of KP enzyme expression in depressed patients.

Further interesting patterns emerge when assessing associations between KP enzyme mRNA levels and circulating KP metabolite concentrations. Intriguingly, the correlation between *IDO1* and the KYN/TRP ratio is absent in the depressed patient group, which suggests KP metabolism is out of step with *IDO1* mRNA levels. It is possible that in depression TRP is metabolised to other KP metabolites at a faster rate, which may influence the association between *IDO1* levels and the KYN/TRP ratio. Peripheral blood TRP concentration is commonly reported as being lower in individuals with depression (Ryan *et al*., 2020). It is possible that a more rapid conversion of tryptophan to QUIN or KYNA takes place in patients with depression influencing the QUIN/KYN and KYNA/KYN ratios and their associations with KP enzyme expression. Moreover, the negative correlations between *IDO1* and the KYNA/QUIN and KYNA/KYN ratios suggest that *IDO1* levels in depression associate with lower activation of the neuroprotective branch of the pathway. Further to this, the negative correlations between (1) *KAT1* mRNA and KYNA metabolite levels and (2) *KAT1* mRNA and the KYNA/KYN ratio are unique to the depressed patient group. This may seem counterintuitive as KAT enzymes are responsible for the conversion of KYN to KYNA, and *KAT1* expression might be expected to positively correlate with KYNA concentrations. This raises a possibility that in depressed patients *KAT1* expression may be adaptive to compensate for reduced KYNA concentrations, although further research is needed to investigate this possibility. However, KAT enzymes also convert 3-HK to XA, a reaction which may take precedence in depression. Further insights will be gained by quantifying enzyme activity.

Multiple transcript variants that encode different isoforms have been identified for *KAT*. pH is important for the functioning of these enzymes. KAT2 has an optimum pH at 7.4, KAT1 at pH 10, KAT3 at pH 9 and KAT4 at pH 8, where KAT2 is considered to preferentially control the pool of KYNA that can be rapidly mobilised in the brain (Wonodi and Schwarcz, 2010; González Esquivel *et al*., 2017). mRNA expression of *KAT2* was also measured in this study, but expression levels were below the limits of detection. It is not possible to assume that *KAT1* is a proxy for all KAT enzymes. *KAT1*, a cytosolic enzyme, is ubiquitously expressed in brain and many other tissues (Fagerberg *et al*., 2014) but with a prominence in the periphery, its expression in blood and tissue compartments outside of the brain are likely to be more influential in regulating the conversion of KYN to KYNA possibly regulating the extent to which KYN may access the brain. The extent of alignment between peripheral and central alterations is however uncertain. A systematic review on brain versus blood kynurenine pathway measures in psychiatric disorders has reported concordance between peripheral and central concentrations of KYN and 3-HK (Skorobogatov *et al*., 2021). Nevertheless, additional research is needed to ascertain the validity of other peripheral pathway measures as proxy for activity within the central nervous system. This does not discount for the possibility that changes in expression of *KAT1* in the brain may also be functionally relevant. Increased activity of KAT1 has been reported in the cerebellum of patients with schizophrenia supporting an NMDA hypofunction hypothesis (Kapoor *et al*., 2006). Clark *et al.* (2016) reported a significant decrease in kynurenine pathway activation in the ventrolateral prefrontal cortex (VLPFC), with decreased levels of QUIN and decreased gene expression of *IDO1*, *IDO2* and *TDO2*. Future studies to measure the expression of all transcripts of KAT (in addition to other kynurenine pathway targets) and correlation of these with enzyme activities in brain from healthy and depressed individuals will enable a more precise identification of the transcripts most relevant to depression.

The altered correlation patterns between KP enzyme, *NR3C1* and *IL-6* mRNA levels observed in depressed patients pre- and post-ECT support stress- and inflammatory-related mechanisms underlying changes to the KP. In depressed patients, *NR3C1* is associated with induction of the KP, favouring the production of metabolites related to oxidative and neurotoxic properties. As neither of the *NR3C1* correlations remained in depressed patients post-ECT, ECT appears to alter associations between *NR3C1* and KP enzyme levels. Peripheral *TDO* expression is restricted to the liver (Stone, 1993), making it impossible to measure mRNA expression of this enzyme in whole blood. As TDO induction is largely mediated by the glucocorticoid receptor (as discussed in a review by O’Farrell and Harkin (2017)), a determination of association between expression of *TDO* with *NR3C1* mRNA levels would be of further interest.

Our analysis of DNA methylation offers additional evidence supporting the involvement of altered gene expression of KP enzymes in depression, since hypermethylation is frequently associated with gene silencing. However, DNA methylation analysis of individuals with a self-reported history of depression is a limitation as self-reported history is not necessarily representative of a clinical depressive episode. Therefore, further investigations should explore the potential impact of epigenetic mechanisms on the gene expression of KP enzymes in depression, requiring concurrent analysis of matched DNA and RNA samples from the same individuals.

A further limitation of the present study is that full blood cell counts were unavailable at the time at which blood was collected for KP analysis. It is possible that alterations in blood cell numbers may be responsible for the reduced expression of KP enzymes observed in this study. The most abundant cell type in blood is the neutrophil, varying from 30 to 70% of the white blood cell count in healthy adults (Palmer *et al*., 2006). Neutrophil count, leucocyte count and neutrophil–lymphocyte ratio are raised in unmedicated depressed patients (Demir *et al*., 2015, Foley *et al*., 2023). As whole blood was used in the present study, detection of mRNA from less abundant cell types (e.g., lymphocytes, macrophages) may be poor. Human neutrophils contain functional IDO, which may play a role in their cytotoxic activity (Ishio *et al*., 2004; Kai *et al*., 2004; Souza-Fonseca-Guimaraes *et al*., 2012). Information regarding *KAT1* expression in neutrophils is lacking. IDO is predominantly expressed in monocytes, though it is also present in T lymphocytes, B-lymphocytes and natural killer cells (Kai *et al*., 2004; De Ravin *et al*., 2010). Flow cytometric analyses have shown that while there is a basal level of expression of IDO1 and KMO in lymphocytes, upregulation of these enzymes does not occur in lymphocytes following IFN-γ stimulation. By contrast, IDO1 and KMO protein show high basal expression in monocytes and are upregulated following IFN-γ treatment (Jones *et al*., 2015). Since blood is a complex tissue that is composed of several different types of cells that all have their own unique functions and gene expression profiles, there is a need for additional studies examining cell type specific gene expression in human peripheral blood.

An additional limitation of this study is that patients with depression were receiving treatment with a range of medications throughout their course of treatment with ECT. Therefore, it was not possible to analyse the effects of individual medications on KP enzyme expression. While there is some evidence that lithium can suppress *IDO1* mRNA transcription, protein expression and activity in primary and immortalised human microglial cells *in vitro* (Göttert *et al*., 2021), data on the effects of antidepressant medication on KP enzyme expression in peripheral cells *in vitro* or *in vivo* are sparse. Indeed, additional large-scale studies are also required to determine if mRNA expression of KP enzymes are altered in blood samples from unmedicated patients with depression compared to healthy controls.

In summary, mRNA levels of KP enzyme targets are prominently associated with plasma KP metabolites and *NR3C1* and *IL-6* mRNA levels in healthy controls and in depressed patients pre- and post-ECT. Associations differ in depressed patients compared to healthy controls and to a lesser extent post-ECT. Further studies are warranted as KP measures could potentially be integrated into stress and immune associated biomarker panels to aid patient stratification at diagnosis and in predicting treatment response to antidepressant therapy.

## Supporting information

Ryan et al. supplementary material 1 Ryan et al. supplementary material

Ryan et al. supplementary material 2 Ryan et al. supplementary material

Ryan et al. supplementary material 3 Ryan et al. supplementary material

## Supplementary material

The supplementary material for this article can be found at https://doi.org/10.1017/neu.2024.34.
