# Supplementary material for: Gene expression of kynurenine pathway enzymes in depression and following electroconvulsive therapy
Source: Acta Neuropsychiatr. 2024 Oct 17;37:e34. doi: 10.1017/neu.2024.34 (PMC13130249; doi:10.1017/neu.2024.34)
Supplement: Ryan et al. supplementary material 1 — Ryan et al. supplementary material [file S0924270824000346sup001.docx]

| **Supplemental Table 1**. KP enzymes in patients with unipolar and bipolar depression pre- and post-ECT | | | | | | |
| --- | --- | --- | --- | --- | --- | --- |
|  | | Polarity | Pre-ECT | Post-ECT | Unadjusted Statistics | Adjusted Statistics |
| *KAT*1^#^ | | Unipolar (n=56) | 1.85 (0.63) | 1.83 (0.58) | *Time: F*_1,72_ = 0.907, *p* = 0.344  *Group:* *F*_1,72_ = 0.181, *p* = 0.672  *Time×Group:* *F*_1,72_ = 0.976, *p* = 0.326 | *Time: F*_1,63_ = 0.006, *p* = 0.937  *Group:* *F*_1,63_ = 0.286, *p* = 0.595  *Time×Group:* *F*_1,63_ = 1.524, *p* = 0.222 |
|  | | Bipolar (n=18) | 1.76 (0.42) | 1.94 (0.53) |  |  |
|  | |  |  |  |  |  |
| *KMO^#^* | | Unipolar (n=56) | 1.90 (0.83) | 1.99 (0.88) | *Time: F*_1,71_ = 1.453, *p* = 0.232  *Group:* *F*_1,71_ = 0.005, *p* = 0.947  *Time×Group:* *F*_1,71_ = 0.134, *p* = 0.715 | *Time: F*_1,62_ = 0.848, *p* = 0.361  *Group:* *F*_1,62_ = 0.146, *p* = 0.704  *Time×Group:* *F*_1,62_ = 0.149, *p* = 0.701 |
|  | | Bipolar (n=17) | 1.78 (0.64) | 2.04 (0.99) |  |  |
|  | |  |  |  |  |  |
| *KYNU* | | Unipolar (n=54) | 0.62 (0.34) | 0.67 (0.31) | *Pre: U=428.50, p = 0.681*  *Post: U=470, p = 0.882*  *Unipolar: Z=861, p = 0.308*  *Bipolar: Z=108, p = 0.136* |  |
|  | | Bipolar (n=17) | 0.55 (0.10) | 0.66 (0.24) |  |  |
|  | |  |  |  |  |  |
|  | |  |  |  |  |  |
| *IDO1^#^* | | Unipolar (n=53) | 4.34 (4.02) | 3.99 (3.97) | *Time: F*_1,68_ = 0.199, *p* = 0.657  *Group:* *F*_1,68_ = 0.118, *p* = 0.732  *Time×Group:* *F*_1,68_ = 0.118, *p* = 0.732 | *Time: F*_1,59_ = 2.731, *p* = 0.104  *Group:* *F*_1,59_ = 0.059, *p* = 0.809  *Time×Group:* *F*_1,59_ = 0.236, *p* = 0.629 |
|  | | Bipolar (n=17) | 3.13 (1.79) | 4.81 (7.91) |  |  |
|  | |  |  |  |  |  |
| *IDO2* | | Unipolar (n=53) | 1.09 (0.71) | 1.45 (1.18) | *Pre: U = 418, p = 0.656*  *Post: U = 423, p = 0.706*  *Unipolar: Z = 885.50, p = 0.132*  *Bipolar: Z = 90, p = 0.523* |  |
|  | | Bipolar (n=17) | 1.05 (0.71) | 1.49 (1.55) |  |  |
|  | Data are presented as mean (SD).  ^#^Log_10_ transformed data used for statistical analysis | | | | | |

| **Supplemental Table 2**. KP enzymes in patients with psychotic and non-psychotic depression pre- and post-ECT | | | | | | |
| --- | --- | --- | --- | --- | --- | --- |
|  | | Psychosis | Pre-ECT | Post-ECT | Unadjusted Statistics | Adjusted Statistics |
| *KAT1* | | Yes (n=19) | 1.94 (0.60) | 1.84 (0.63) | *Pre: U* = 594, *p* = 0.376  *Post: U* = 479, *p* = 0.590  *Psychotic: Z* = 88, *p* = 0.778  *Non-psychotic: Z* = 925, *p* = 0.194 |  |
|  | | No (n=55) | 1.79 (0.58) | 1.86 (0.56) |  |  |
|  | |  |  |  |  |  |
|  | |  |  |  |  |  |
| *KMO^#^* | | Yes (n=19) | 1.89 (0.80) | 2.00 (1.05) | *Time: F*_1,71_ = 0.635, *p* = 0.428  *Group:* *F*_1,71_ = 0.0003, *p* = 0.985  *Time×Group:* *F*_1,71_ = 0.277, *p* = 0.600 | *Time: F*_1,62_ = 0.848, *p* = 0.361  *Group:* *F*_1,62_ = 0.034, *p* = 0.854  *Time×Group:* *F*_1,62_ = 1.835, *p* = 0.180 |
|  | | No (n=54) | 1.86 (0.80) | 2.00 (0.86) |  |  |
|  | |  |  |  |  |  |
| *KYNU* | | Yes (n=18) | 0.67 (0.20) | 0.65 (0.30) | *Pre: U* = 628, *p* = 0.046  *Post: U* = 444.50, *p* = 0.668  *Psychotic: Z* = 65, *p* = 0.372  *Non-psychotic: Z* = 970, *p* = 0.024 |  |
|  | | No (n=53) | 0.58 (0.32) | 0.67 (0.30) |  |  |
|  | |  |  |  |  |  |
|  | |  |  |  |  |  |
| *IDO1* | | Yes (n=18) | 5.26 (5.09) | 4.00 (4.17) | *Pre: U* = 543, *p* = 0.314  *Post: U* = 462, *p* = 0.936  *Psychotic: Z* = 64, *p* = 0.349  *Non-psychotic: Z* = 673, *p* = 0.884 |  |
|  | | No (n=52) | 3.63 (2.92) | 4.26 (5.48) |  |  |
|  | |  |  |  |  |  |
| *IDO2* | | Yes (n=18) | 1.21 (0.93) | 1.50 (1.29) | *Pre: U* = 488, *p* = 0.788  *Post: U* = 484, *p* = 0.830  *Psychotic: Z* = 93, *p* = 0.744  *Non-psychotic: Z* = 875, *p* = 0.090 |  |
|  | | No (n=52) | 1.03 (0.61) | 1.45 (1.27) |  |  |
|  | |  |  |  |  |  |
|  | |  |  |  |  |  |
|  | Data are presented as mean (SD).  ^#^Log_10_ transformed data used for statistical analysis | | | | | |

| **Supplemental Table 3**. KP enzymes in remitters and non-remitters pre- and post-ECT | | | | | | |
| --- | --- | --- | --- | --- | --- | --- |
|  | | Remitter | Pre-ECT | Post-ECT | Unadjusted Statistics | Adjusted Statistics |
| *KATI^#^* | | Yes (n=39) | 1.74 (0.55) | 1.75 (0.52) | *Time: F*_1,72_ = 0.27, *p* = 0.605  *Group:* *F*_1,72_ = 3.303, *p* = 0.073  *Time×Group:* *F*_1,72_ = 0.011, *p* = 0.918 | *Time: F*_1,62_ = 0.023, *p* = 0.879  *Group:* *F*_1,62_ = 2.14, *p* = 0.15  *Time×Group:* *F*_1,62_ = 0.168, *p* = 0.684 |
|  | | No (n=35) | 1.93 (0.62) | 1.98 (0.61) |  |  |
|  | |  |  |  |  |  |
|  | |  |  |  |  |  |
| *KMO^#^* | | Yes (n=39) | 1.78 (0.74) | 2.05 (1.04) | *Time: F*_1,71_ = 1.255, *p* = 0.266  *Group:* *F*_1,71_ = 0.257, *p* = 0.614  *Time×Group:* *F*_1,71_ = 1.439, *p* = 0.234 | *Time: F*_1,61_ = 0.431, *p* = 0.514  *Group:* *F*_1,61_ = 0.049, *p* = 0.825  *Time×Group:* *F*_1,61_ = 1.073, *p* = 0.304 |
|  | | No (n=34) | 1.97 (0.84) | 1.94 (0.73) |  |  |
|  | |  |  |  |  |  |
| *KYNU^#^* | | Yes (n=39) | 0.63 (0.37) | 0.65 (0.33) | *Time: F*_1,69_ = 4.84, *p* = 0.031  *Group:* *F*_1,69_ = 0.150, *p* = 0.700  *Time×Group:* *F*_1,69_ = 0.757, *p* = 0.387 | *Time: F*_1,59_ = 0.033, *p* = 0.855  *Group:* *F*_1,59_ = 0.439, *p* = 0.51  *Time×Group:* *F*_1,59_ = 1.223, *p* = 0.273 |
|  | | No (n=32) | 0.57 (0.17) | 0.68 (0.25) |  |  |
|  | |  |  |  |  |  |
| *IDO1^#^* | | Yes (n=38) | 3.98 (3.86) | 4.97 (6.54) | *Time: F*_1,68_ = 0.747, *p* = 0.391  *Group:* *F*_1,68_ = 0.769, *p* = 0.384  *Time×Group:* *F*_1,68_ = 2.284, *p* = 0.135 | *Time: F*_1,58_ = 1.97, *p* = 0.166  *Group:* *F*_1,58_ = 0.313, *p* = 0.578  *Time×Group:* *F*_1,58_ = 1.222, *p* = 0.273 |
|  | | No (n=32) | 4.12 (3.40) | 3.26 (2.52) |  |  |
|  | |  |  |  |  |  |
| *IDO2* | | Yes (n=39) | 0.99 (0.57) | 1.22 (1.10) | *Pre: U* = 642, *p* = 0.657  *Post: U* = 765, *p* = 0.058  *Remitter: Z* = 435.50, *p* = 0.525  *Non-remitter: Z* = 0.328, *p* = 0.117 |  |
|  | | No (n=31) | 1.19 (0.84) | 1.77 (1.41) |  |  |
|  | |  |  |  |  |  |
|  | Data are presented as mean (SD).  ^#^Log_10_ transformed data used for statistical analysis | | | | | |

| **Supplemental Table 4**. KP enzymes in responders and non-responders pre- and post-ECT | | | | | | |
| --- | --- | --- | --- | --- | --- | --- |
|  | | Responder | Pre-ECT | Post-ECT | Unadjusted Statistics | Adjusted Statistics |
| *KATI* | | Yes (n=45) | 1.77 (0.53) | 1.80 (0.55) | *Pre: U* = 715*, p* = 0.489  *Post: U* = 743, *p* = 0.316  *Responder: Z* = 567.50, *p* = 0.572  *Non-responder: Z* = 250, *p* = 0.482 |  |
|  | | No (n=29) | 1.92 (0.66) | 1.94 (0.60) |  |  |
|  | |  |  |  |  |  |
|  | |  |  |  |  |  |
| *KMO^#^* | | Yes (n=45) | 1.79 (0.71) | 2.06 (0.99) | *Time: F*_1,71_ = 0.727, *p* = 0.397  *Group:* *F*_1,71_ = 0.046, *p* = 0.831  *Time×Group:* *F*_1,71_ = 1.926, *p* = 0.170 | *Time: F*_1,61_ = 0.254, *p* = 0.616  *Group:* *F*_1,61_ = 0.019, *p* = 0.891  *Time×Group:* *F*_1,61_ = 2.747, *p* = 0.103 |
|  | | No (n=28) | 1.99 (0.91) | 1.90 (0.74) |  |  |
|  | |  |  |  |  |  |
| *KYNU^#^* | | Yes (n=45) | 0.63 (0.35) | 0.68 (0.34) | *Time: F*_1,69_ = 4.58, *p* = 0.036  *Group:* *F*_1,69_ = 0.141, *p* = 0.708  *Time×Group:* *F*_1,69_ = 0.146, *p* = 0.703 | *Time: F*_1,59_ = 0.002, *p* = 0.963  *Group:* *F*_1,59_ = 0.010, *p* = 0.921  *Time×Group:* *F*_1,59_ = 0.000007, *p* = 0.984 |
|  | | No (n=26) | 0.56 (0.17) | 0.65 (0.21) |  |  |
|  | |  |  |  |  |  |
| *IDO1^#^* | | Yes (n=44) | 3.94 (3.77) | 4.57 (6.18) | *Time: F*_1,68_ = 0.853, *p* = 0.359  *Group:* *F*_1,68_ = 0.047, *p* = 0.830  *Time×Group:* *F*_1,68_ = 0.725, *p* = 0.398 | *Time: F*_1,58_ = 2.16, *p* = 0.147  *Group:* *F*_1,58_ = 0.349, *p* = 0.557  *Time×Group:* *F*_1,58_ = 0.451, *p* = 0.505 |
|  | | No (n=26) | 4.22 (3.46) | 3.55 (2.61) |  |  |
|  | |  |  |  |  |  |
| *IDO2* | | Yes (n=45) | 1.03 (0.62) | 1.30 (1.19) | *Pre: U* = 569*, p* = 0.936  *Post: U* = 681*, p* = 0.146  *Responder: Z =* 581.50*, p =* 0.470  *Non-remitter: Z =* 222*, p =* 0.109 |  |
|  | | No (n=25) | 1.16 (0.84) | 1.75 (1.38) |  |  |
|  | |  |  |  |  |  |
|  | Data are presented as mean (SD).  ^#^Log_10_ transformed data used for statistical analysis | | | | | |

| **Supplemental Table 5.** Correlations between *IDO2* and HAM-D24 scores | | | |
| --- | --- | --- | --- |
|  | Baseline *IDO2* & Baseline HAM-D24 | Baseline *IDO2* & ΔHAM-D24 | Δ*IDO2* &  ΔHAM-D24 |
| *Polarity* |  |  |  |
| Unipolar (*n* = 52) | *ρ* = -0.212, *p* = 0.127 | *ρ* = 0.143, *p* = 0.311 | *ρ* = 0.014, *p* = 0.924 |
| Bipolar (*n* = 17) | *r* = -0.275, *p* = 0.286 | *r* = 0.117, *p* = 0.654 | *ρ* = -0.264, *p* = 0.306 |
| *Psychosis* |  |  |  |
| Yes (*n* = 18) | *ρ* = -0.584, ***p* = 0.011** | *ρ* = 0.458, *p* = 0.187 | *ρ* = -0.346, *p* = 0.160 |
| No (*n* = 51) | *ρ* = -0.074*, p* = 0.603 | *ρ* = 0.064, *p* = 0.657 | *ρ* = 0.055, *p* = 0.699 |
| *Response* |  |  |  |
| Yes (*n* = 45) | *ρ* = -0.107, *p* = 0.485 | *ρ* = 0.144, *p* = 0.346 | *ρ* = -0.317, *p* = 0.034 |
| No (*n* = 24) | *ρ* = -0.349, *p* = 0.088 | *ρ* = 0.099, *p* = 0.647 | *r* = -0.257, *p* = 0.226 |
| *Remission* |  |  |  |
| Yes (*n* = 39) | *ρ* = -0.062, *p* = 0.709 | *r* = 0.113, *p* = 0.495 | *ρ* = -0.254, *p* = 0.118 |
| No (*n* = 30) | *ρ* = -0.417, ***p* = 0.020** | *ρ* = 0.078, *p* = 0.684 | *r* =  *ρ* = -0.225, *p* = 0.232 |
| Abbreviations: HAM-D24, Hamilton depression rating scale, 24-item version; ΔHAM-D24, change in Hamilton depression rating scale, 24-item version score. | | | |

| **Supplemental Table 6.** Correlations between *KATI* and HAM-D24 scores | | | |
| --- | --- | --- | --- |
|  | Baseline *KATI* & Baseline HAM-D24 | Baseline *KATI* & ΔHAM-D24 | Δ*KATI* &  ΔHAM-D24 |
| *Polarity* |  |  |  |
| Unipolar (*n* = 55) | *ρ* = -0.044, *p* = 0.747 | *r =* 0.095, *p* = 0.492 | *r =* 0.035, *p* = 0.798 |
| Bipolar (*n* = 18) | *r* = 0.478, *p* = 0.045 | *r* = -0.013, *p* = 0.960 | *r* = -0.195, *p* = 0.439 |
| *Psychosis* |  |  |  |
| Yes (*n* = 19) | *r* = 0.006, *p* = 0.982 | *r* = 0.442, *p* = 0.058 | *r* = -0.362, *p* = 0.127 |
| No (*n* = 54) | *ρ* = 0.010*, p* = 0.944 | *ρ* = -0.054, *p* = 0.699 | *r* = 0.160, *p* = 0.248 |
| *Response* |  |  |  |
| Yes (*n* = 45) | *ρ* = 0.228, *p* = 0.131 | *ρ* = -0.163, *p* = 0.284 | *ρ* = -0.119, *p* = 0.435 |
| No (*n* = 28) | *ρ* = -0.224, *p* = 0.242 | *ρ* = 0.091, *p* = 0.646 | *r* = 0.077, *p* = 0.697 |
| *Remission* |  |  |  |
| Yes (*n* = 39) | *ρ* = 0.260, *p* = 0.110 | *r* = -0.103, *p* = 0.531 | *r* = -0.015, *p* = 0.926 |
| No (*n* = 34) | *r* = -0.191, *p* = 0.271 | *r* = 0.039, *p* = 0.827 | *ρ* = 0.041, *p* = 0.819 |
| Abbreviations: HAM-D24, Hamilton depression rating scale, 24-item version; ΔHAM-D24, change in Hamilton depression rating scale, 24-item version score. | | | |

| **Supplemental Table 7.**  Correlations between KYNU and HAM-D24 scores | | | |
| --- | --- | --- | --- |
|  | Baseline *KYNU* & Baseline HAM-D24 | Baseline *KYNU* & ΔHAM-D24 | Δ*KYNU* &  ΔHAM-D24 |
| *Polarity* |  |  |  |
| Unipolar (*n* = 53) | *ρ* = 0.084, *p* = 0.544 | *ρ* = *-*0.167, *p* = 0.233 | *ρ* = 0.240, *p* = 0.084 |
| Bipolar (*n* = 17) | *ρ* = -0.041, *p* = 0.875 | *ρ* = -0.273, *p* = 0.289 | *r* = -0.416, *p* = 0.097 |
| *Psychosis* |  |  |  |
| Yes (*n* = 18) | *r* = 0.367, *p* = 0.135 | *r* = -0.472, *p* = 0.048 | *ρ* = -0.017, *p* = 0.906 |
| No (*n* = 52) | *ρ* = -0.168*, p* = 0.229 | *ρ* = -0.025, *p* = 0.861 | *r* = 0.232, *p* = 0.354 |
| *Response* |  |  |  |
| Yes (*n* = 45) | *ρ* = 0.036, *p* = 0.816 | *ρ* = -0.109, *p* = 0.476 | *ρ* = -0.032, *p* = 0.836 |
| No (*n* = 25) | *r* = 0.121, *p* = 0.557 | *r* = -0.253, *p* = 0.222 | *r* = 0.435, *p* = 0.030 |
| *Remission* |  |  |  |
| Yes (*n* = 39) | *ρ* = 0.013, *p* = 0.936 | *ρ* = -0.121, *p* = 0.464 | *r* = 0.003, *p* = 0.986 |
| No (*n* = 31) | *r* = 0.173, *p* = 0.345 | *r* = -0.306, *p* = 0.094 | *ρ* = 0.165, *p* = 0.376 |
|  |  |  |  |
| Abbreviations: HAM-D24, Hamilton depression rating scale, 24-item version; ΔHAM-D24, change in Hamilton depression rating scale, 24-item version score. | | | |

| **Supplemental Table 8.** Correlation data used to generate Figure 1. | | |
| --- | --- | --- |
| KP metabolite correlations with other KP metabolites | | |
| ***Healthy control cohort*** | **Correlation coefficient (ρ)** | **P value** |
| TRP & KYN | 0.422 | 0.016 |
| TRP & 3-HK | 0.504 | 0.003 |
| TRP & KYNA | 0.561 | 0.001 |
| TRP & XA | 0.492 | 0.004 |
| TRP & 3-HAA | 0.556 | 0.001 |
| TRP & PIC | 0.518 | 0.002 |
| 3-HK & KYN | 0.744 | 0.000001 |
| KYN & KYNA | 0.702 | 0.000007 |
| KYN & 3-HAA | 0.691 | 0.000012 |
| KYN & PIC | 0.455 | 0.009 |
| KYN & QUIN | 0.766 | 3.32 × 10^-7^ |
| 3-HK & KYNA | 0.746 | 9.46 ×10^-7^ |
| 3-HK & XA | 0.709 | 0.000006 |
| 3-HK & 3-HAA | 0.771 | 2.47 × 10^-7^ |
| 3-HK & PIC | 0.653 | 0.00005 |
| 3-HK & QUIN | 0.655 | 0.000047 |
| KYNA & XA | 0.752 | 6.94 × 10^-7^ |
| KYNA & 3-HAA | 0.618 | 0.00016 |
| KYNA & PIC | 0.612 | 0.000199 |
| KYNA & QUIN | 0.49 | 0.004 |
| XA & 3-HAA | 0.689 | 0.000013 |
| XA & PIC | 0.769 | 2.77 × 10^-7^ |
| XA & QUIN | 0.407 | 0.021 |
| 3-HAA & PIC | 0.571 | 0.001 |
| 3-HAA & QUIN | 0.645 | 0.000066 |
| PIC & QUIN | 0.405 | 0.022 |
|  |  |  |
| ***Depressed pre-ECT cohort*** | **ρ** | **P value** |
| TRP & KYN | 0.513 | 0.000011 |
| TRP & KYNA | 0.314 | 0.011 |
| TRP & XA | 0.458 | 0.000123 |
| TRP & 3-HAA | 0.544 | 0.000003 |
| TRP & PIC | 0.283 | 0.022 |
| KYN & 3-HK | 0.696 | 1.23 × 10^-10^ |
| KYN & KYNA | 0.609 | 7.27 × 10^-8^ |
| KYN & XA | 0.57 | 7.31 × 10^-7^ |
| KYN & 3-HAA | 0.663 | 1.78 × 10^-9^ |
| KYN & PIC | 0.265 | 0.033 |
| KYN & QUIN | 0.79 | 5.38 × 10^-15^ |
| 3-HK & KYNA | 0.437 | 0.00027 |
| 3-HK & XA | 0.381 | 0.002 |
| 3-HK & 3-HAA | 0.419 | 0.001 |
| 3-HK & QUIN | 0.574 | 5.91 × 10^-7^ |
| KYNA & XA | 0.779 | 2.07 × 10^-14^ |
| KYNA & 3-HAA | 0.697 | 1.11 × 10^-10^ |
| KYNA & PIC | 0.567 | 8.61 × 10^-7^ |
| KYNA & QUIN | 0.41 | 0.001 |
| XA & 3-HAA | 0.819 | 7.60 × 10^-17^ |
| XA & PIC | 0.576 | 5.18 × 10^-7^ |
| XA & QUIN | 0.39 | 0.001 |
| 3-HAA & PIC | 0.585 | 3.05 × 10^-7^ |
| 3-HAA & QUIN | 0.474 | 0.000067 |
|  |  |  |
| ***Depressed post-ECT cohort*** | **ρ** | **P value** |
| TRP & KYN | 0.332 | 0.010 |
| TRP & KYNA | 0.381 | 0.003 |
| TRP & XA | 0.497 | 0.000062 |
| TRP & HAA | 0.432 | 0.001 |
| TRP & PIC | 0.367 | 0.004 |
| KYN & 3-HK | 0.692 | 1.29 × 10^-9^ |
| KYN & KYNA | 0.683 | 2.56 × 10^-9^ |
| KYN & XA | 0.483 | 0.00011 |
| KYN & 3-HAA | 0.6 | 5.21 × 10^-7^ |
| KYN & PIC | 0.261 | 0.046 |
| KYN & QUIN | 0.855 | 6.65 × 10^-18^ |
| 3-HK & KYNA | 0.589 | 9.14 × 10^-7^ |
| 3-HK & XA | 0.477 | 0.000134 |
| 3-HK & 3-HAA | 0.593 | 7.39 × 10^-7^ |
| 3-HK & PIC | 0.273 | 0.037 |
| 3-HK & QUIN | 0.718 | 1.59 × 10^-10^ |
| KYNA & XA | 0.711 | 2.75 × 10^-10^ |
| KYNA & 3-HAA | 0.669 | 6.74 × 10^-9^ |
| KYNA & PIC | 0.481 | 0.00012 |
| KYNA & QUIN | 0.576 | 0.000002 |
| XA & 3-HAA | 0.779 | 3.90 × 10-^13^ |
| XA & PIC | 0.512 | 0.000034 |
| XA & QUIN | 0.466 | 0.0002 |
| 3-HAA & PIC | 0.57 | 0.000002 |
| 3-HAA & QUIN | 0.609 | 3.01 × 10^-7^ |
|  |  |  |
| KP enzyme mRNA expression correlations with other KP enzymes | | |
| ***Healthy control cohort*** | **ρ** | **P value** |
| *KMO* & *KYNU* | 0.533 | 0.000048 |
| *IDO1* & *IDO2* | 0.481 | 0.000266 |
|  |  |  |
| ***Depressed pre-ECT cohort*** | **ρ** | **P value** |
| *KMO* & *KYNU* | 0.457 | 0.000145 |
| *KMO* & *KAT1* | 0.295 | 0.011 |
| *IDO1* & *IDO2* | 0.300 | 0.012 |
| *IDO1* & *KMO* | 0.477 | 0.000029 |
| *IDO1* & *KAT1* | 0.288 | 0.015 |
| *IDO2* & *KAT1* | 0.328 | 0.006 |
| ***Depressed post-ECT cohort*** | **ρ** | **P value** |
| *KMO* & *KYNU* | 0.595 | 4.582 × 10^-8^ |
| *KMO* & *KAT1* | 0.317 | 0.006 |
| *IDO1* & *IDO2* | 0.311 | 0.009 |
| *IDO2* & *KAT1* | 0.270 | 0.024 |
|  |  |  |
| KP enzyme mRNA expression correlations with KP metabolites | | |
| ***Healthy control cohort*** | **ρ** | **P value** |
| *IDO1* & KYN/TRP | 0.382 | 0.031 |
| *KMO* & 3-HK | 0.402 | 0.023 |
| *KMO* & PIC | 0.350 | 0.049 |
| *KAT1* & PIC | 0.372 | 0.036 |
|  |  |  |
| ***Depressed pre-ECT cohort*** | **ρ** | **P value** |
| *IDO1* & KYNA/KYN | -0.324 | 0.010 |
| *IDO1* & KYNA/QUIN | -0.368 | 0.003 |
| *KYNU* & TRP | -0.250 | 0.045 |
| *KYNU* & KYN | -0.258 | 0.038 |
| *KYNA* & KAT1 | -0.265 | 0.033 |
| *KAT1* & KYNA/KYN | -0.305 | 0.013 |
|  |  |  |
| ***Depressed post-ECT cohort*** | **ρ** | **P value** |
| *IDO1* & KYNA/KYN | -0.323 | 0.014 |
| *IDO1* & KYNA/QUIN | -0.381 | 0.003 |
| *IDO2* & KYNA/KYN | -0.326 | 0.013 |
| *IDO2* & KYNA/QUIN | -0.265 | 0.046 |
|  |  |  |
| KP enzyme mRNA expression correlations with markers of stress and inflammation | | |
| ***Healthy control cohort*** | **ρ** | **P value** |
| *KMO* & *IL-6* | 0.327 | 0.017 |
| *KAT1* & *IL-6* | 0.296 | 0.03 |
| *IDO2* & *GILZ** | 0.376 | 0.006 |
| *IDO2* & *NR3C1* | 0.318 | 0.02 |
| *KYNU* & *FKBP5** | -0.306 | 0.027 |
| *KAT1* & *FKBP5** | 0.273 | 0.044 |
| *KAT1* & *NR3C1* | 0.559 | 0.000009 |
|  |  |  |
| ***Depressed pre-ECT cohort*** | **ρ** | **P value** |
| *IDO1* & *IL-6* | 0.312 | 0.009 |
| *IDO2* & *IL-6* | 0.294 | 0.014 |
| *KMO* & *IL-6* | 0.511 | 0.000004 |
| *KAT1* & *IL-6* | 0.327 | 0.005 |
| *IDO1* & *NR3C1* | 0.279 | 0.019 |
| *IDO2* & *NR3C1* | 0.347 | 0.003 |
| *KMO* & *NR3C1* | 0.275 | 0.018 |
| *KAT1* & *NR3C1* | 0.492 | 0.000009 |
|  |  |  |
| ***Depressed post-ECT cohort*** | **ρ** | **P value** |
| IDO2 & *NR3C1* | 0.315 | 0.008 |
| *KYNU* & *GILZ** | 0.305 | 0.01 |
| *KAT1* & *GILZ** | 0.238 | 0.042 |
| *KAT1* & *NR3C1* | 0.473 | 0.000021 |
| *KMO* & *IL-6* | 0.509 | 0.000005 |
| *Denotes a correlation that was not included in Figure 1 in order to improve the clarity of the schematic. | | |

**Supplementary Table 9.** KP enzyme DNA methylation levels in individuals with a history of depression compared to individuals without a history of depression

|  | | | |
| --- | --- | --- | --- |
| **Probe ID** | **Gene annotation from GREAT (distance from TSS)** | **Mean DNA methylation Differences (%)** | **Adjusted P value †** |
| cg13263723 | KYNU (-330445), LRP1B (-415480) | -1% | 0.027 |
| cg21542308 | IDO2 (+61305), C8orf4 (-157208) | 2% | 0.009 |
| cg08465774 | IDO1 (+429) | 0% | 0.822 |
| cg00606312 | KMO (+183) | 0% | 0.206 |
| TSS: Transcriptional start site; † adjusted for history of an inflammatory disorder, age, Sex, anti-depressant use, chip and estimated blood cell composition | | | |
|  |  |  |  |
